# Oxidative degradation of polyamines by serum supplement causes cytotoxicity on cultured cells

**DOI:** 10.1038/s41598-018-28648-8

**Published:** 2018-07-10

**Authors:** Linlin Wang, Ying Liu, Cui Qi, Luyao Shen, Junyan Wang, Xiangjun Liu, Nan Zhang, Tao Bing, Dihua Shangguan

**Affiliations:** 1 Department Beijing National Laboratory for Molecular Sciences, Key Laboratory of Analytical Chemistry for Living Biosystems, CAS Research/Education Center for Excellence in Molecular Sciences, Institute of Chemistry, Chinese Academy of Sciences, Beijing, 100190 China; 2 University of the Chinese Academy of Sciences, Beijing, 100049 China; 3 CAS Center for Excellence in Nanoscience, National Center for Nanoscience and Technology, Beijing, 100190 China

## Abstract

Serum is a common supplement for cell culture due to it containing the essential active components for the growth and maintenance of cells. However, the knowledges of the active components in serum are incomplete. Apart from the direct influence of serum components on cultured cells, the reaction of serum components with tested drugs cannot be ignored, which usually results in the false conclusion on the activity of the tested drugs. Here we report the toxicity effect of polyamines (spermidine and spermine) on cultured cells, especially on drug-resistant cancer cell lines, which resulted from the oxidative degradation of polyamines by amine oxidases in serum supplement. Upon adding spermidine or spermine, high concentration of H_2_O_2,_ an enzyme oxidation product of polyamines, was generated in culture media containing ruminant serum, such as fetal bovine serum (FBS), calf serum, bovine serum, goat serum or horse serum, but not in the media containing human serum. Drug-resistant cancer cell lines showed much higher sensitivity to the oxidation products of polyamines (H_2_O_2_ and acrolein) than their wild cell lines, which was due to their low antioxidative capacity.

## Introduction

Cell culture is a widely used tool to study physiological, biological and pharmacological activities *in vitro*, as well as to produce biological components, such as proteins, hormones and vaccines. This method is fast, cheap, reproducible, and greatly reduces the use of experimental animals. Serum from animals or human is a common supplement for the culture of eukaryotic cells due to it containing a large number of active components, like growth factors, enzymes, hormones, *etc*., which are essential for the growth and maintenance of cells^1–3^. Fetal bovine serum (FBS) is the most commonly used serum in cell culture, because it is rich in fetal growth factors and hormones that stimulate cellular proliferation and maintenance. Although FBS has been used for more than 50 years, the knowledge is incomplete regarding the serum components and their influence on cultured cells^4,5^. Apart from the unknown influence of serum components on the cultured cells directly, the effects of enzymes in serum on the tested drugs should be paid much more attention, otherwise it may result in the false positive or negative results, consequently lead to misleading conclusions. In previous work, we have shown that the cytotoxicity activity of guanine-rich oligonucleotides is attributed to the cytotoxicity of guanine derivatives derived from degradation of oligonucleotides by nuclease in FBS, but not attributed to the direct action of oligonucleotides on cells as reported elsewhere^6^.

As ubiquitous endogenous metabolites, polyamines are essential organic compounds for cell growth and proliferation. Putrescine, spermidine and spermine, existing normally in millimolar concentration range in nucleus^7^, are the mainly naturally occurring polyamines in mammalian cells^8^. Polyamines are involved in a wide variety of cellular processes: participating in the regulation of gene expression and enzyme activity, activating DNA synthesis, facilitating the interaction of DNA and protein, as well as protecting DNA molecules from putative damaging agents^7,9^. Among these polyamines, spermine is reported to possess the highest biological activity^10^. Under physiologic ionic and pH conditions, polyamines are multivalent cations with aliphatic hydrocarbon chains separating the charges^11^, and hence negatively charged nucleic acids, including DNA and RNA, are their prime targets of interaction, and consequently regulate the structures of DNA, condense DNA molecules to a liquid crystalline phase^12^, and enhance the activity of G-quadruplex/hemin DNAzymes *in vitro*^13^. Many evidences suggest that polyamines play important roles as modulators of nucleic acid structure^14^.

The intracellular polyamines are maintained at a correct level through the subtle coordination and regulation of biosynthesis, transport, and catabolism. In mammalian cells, polyamine catabolism occurs through two distinct pathways with the help of three kinds of enzymes: spermidine/spermine N1-acetyltransferase, N1-acetylpolyamine oxidase and spermine oxidase^15^ (Fig. S1). Polyamine catabolism can generate cytotoxic metabolites such as hydrogen peroxide (H_2_O_2_) and aldehydes *in situ*, thereafter inducing apoptosis, necrosis, inhibition of cell proliferation, and inhibition of DNA and protein synthesis^7,16,17^. Polyamines are also the substrates of various kinds of amine oxidases, such as monoamine oxidases, diamine oxidases, polyamine oxidases and copper containing amine oxidases. These enzymes catalyze the oxidative deamination of polyamines to generate the reaction products H_2_O_2_ and aldehyde(s) that are able to induce cell death in several cultured human tumor cell lines^18–23^. Therefore, polyamines and amine oxidases are also considered to have potential in therapeutic applications^15,17^.

Here, we describe the cytotoxicity of polyamines and their degradation products on different cell lines. The generation of H_2_O_2_ (one of the degradation product of polyamines) in culture media containing different serums was measured after addition of spermidine and spermine. The degradation products of spermidine and spermine resulted from the amine oxidases in serum were found to be the main reason of the cytotoxicity of spermidine and spermine.

## Results and Discussion

### The toxicity effect of polyamines on cultured cells

**Figure 1:**
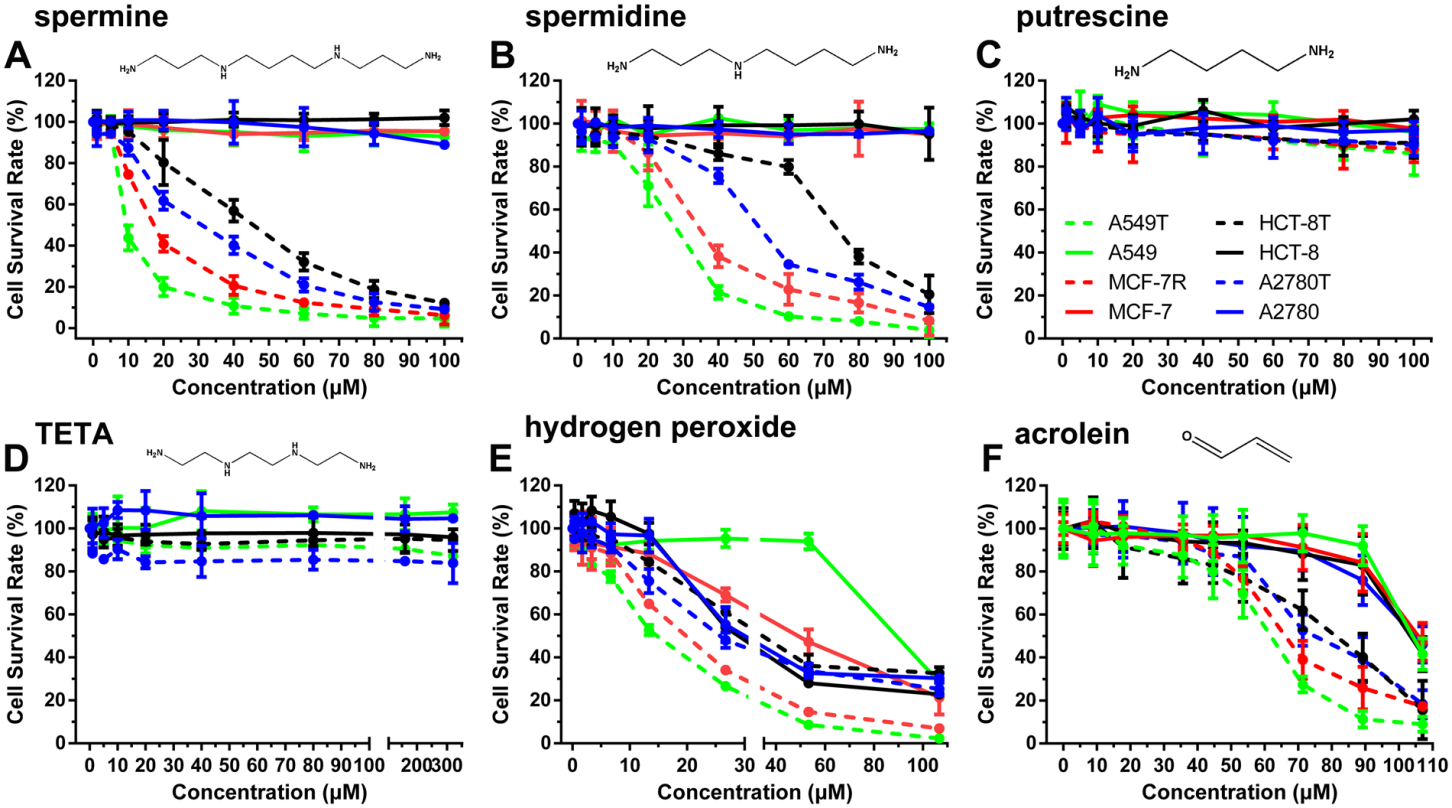
Dose-dependent cytotoxicity effects of polyamines, TETA, hydrogen peroxide and acrolein on cells. A549, A549T, MCF-7, MCF-7R, HCT-8, HCT-8T, A2780 and A2780T cells were respectively incubated with different concentration of (**A**) spermine, (**B**) spermidine, (**C**) putrescine, (**D**) TETA, (**E**) H_2_O_2_, and (**F**) acrolein for 48 h at 37 °C. Each point represents the mean value of the results of 2 to 4 wells of three or four experiments. The error bars indicate ± S.D., when not show, S.D. was smaller than the symbol.

Polyamines (including spermine analogues) have been reported to have cytotoxicity effect on different cells^24–27^. Different mechanisms of action have been reported, such as inducing gene overexpression^28^, telomerase inhibiting^29,30^ and cytotoxicity of the metabolites generated by amine oxidases in cells^31,32^. In order to understand the real mechanism, we tested the cytotoxicity activity of four polyamines (putrescine, spermidine, spermine and triethylenetetramine (TETA)) on eight cell lines *in vitro*, including four cancer cell lines (A549, MCF-7, HCT-8 and A2780) and their drug-resistant sublines (A549T, MCF-7R, HCT-8T and A2780T). As shown in Fig. 1A–D, after treated with spermine and spermidine for 48 h, a dose-dependent cytotoxicity effect on all four drug-resistant cell lines was observed; whereas no significant effect was observed on four wild cell lines. Putrescine and TETA did not show significant cytotoxicity effect on all the tested cell lines. Since TETA, an unnatural compound with a structure similar to spermine, and putrescine are not the substrates of amine oxidases; above suggests that the cytotoxicity effects may not be caused by spermine and spermidine directly, but may be related to the metabolites of polyamines.

**Figure 2:**
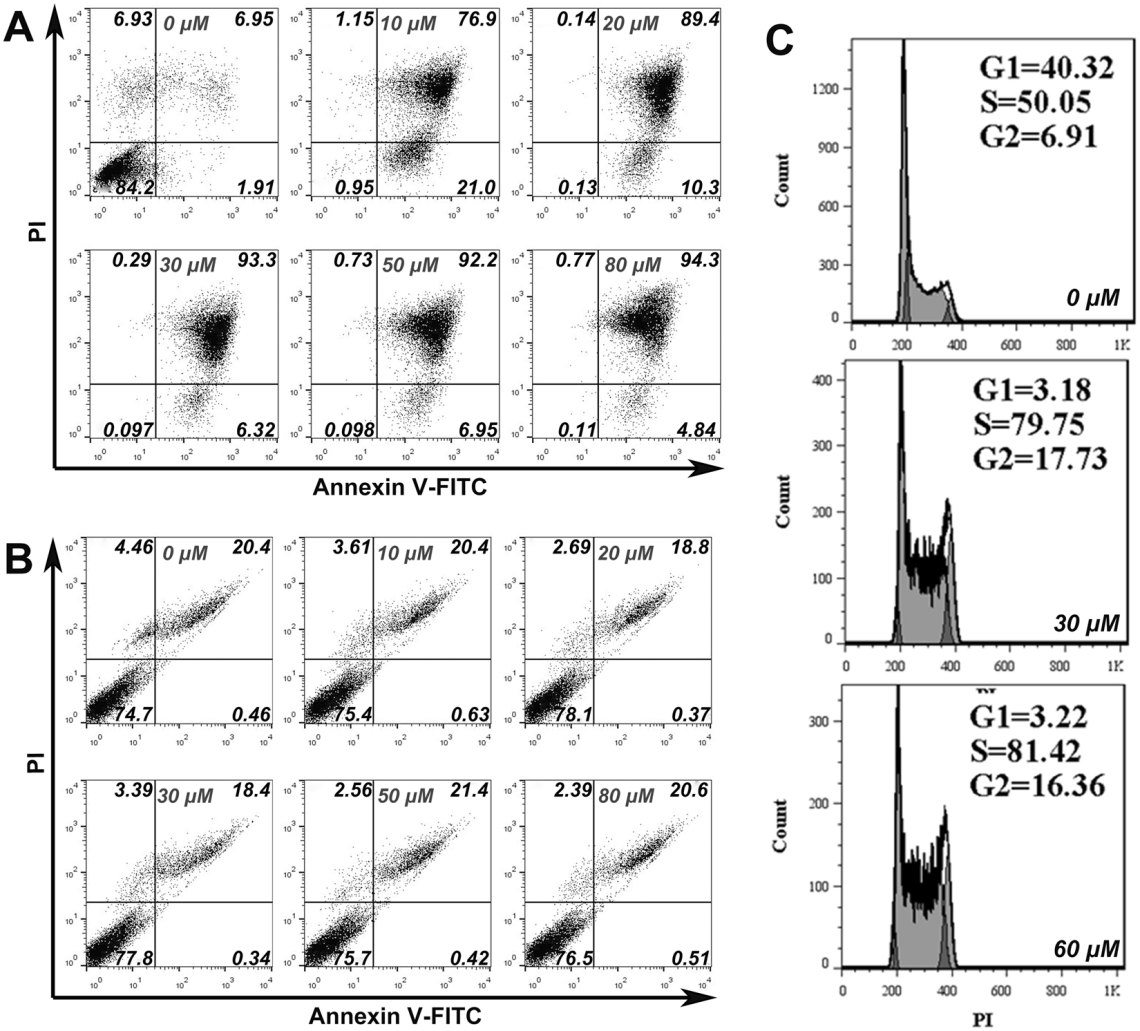
Cell apoptosis and cell cycle assay. (**A**,**B**) Cell apoptosis assay of A549T cells after treated with spermine at 37 °C for 6 h in RPMI1640 with 10% FBS (**A**) and FBS-free RPMI1640 (**B**) respectively. The concentration of spermine respectively is 0, 10, 20, 30, 50 and 80 μM. Cells were double-stained by Annexin V-FITC and PI. Crossing gates divided the dot plots into four quadrants. Dots in lower right quadrants (Annexin V^+^ and PI^−^) indicate early apoptosis cells; dots in upper right quadrants (Annexin V^+^ and PI^+^) indicate late apoptosis cells or dead cells. The numbers indicate the percentage of cells in the corresponding quadrants. (**C**) The cell cycle profiles analysis of A549T treated with spermine. The concentration of spermine respectively is 0, 30 and 60 μM. Quantification of G1, S and G2 phase percentage  was performed by FlowJo (Treestar, San Caros, USA). Results are single representative of three independent experiments.

Since spermine showed stronger cytotoxicity activity on drug-resistant cells over that of spermidine, and an taxol-resistant A549 subline (A549T) was the most sensitive cells to spermine and spermidine among these drug-resistant sublines; the apoptosis assay and cell cycle assay of A549T cells after treatment with spermine were therefore performed. As shown in Fig. 2A, spermine significantly induced cell apoptosis and death in media containing FBS, and caused S phase arrest of A549T cells (Fig. 2C). However, spermine did not cause significant cell apoptosis and death in the media without FBS (Fig. 2B), further suggesting that the cytotoxicity activity of spermine was related to FBS.

It has previously been reported that the enzymatic oxidation products of spermine generated by purified bovine serum amine oxidase show a higher cytotoxicity to several drug-resistant cell lines than their wild type cell lines^33^, such as Chinese hamster ovary cells^18–20^, melanoma cells line (M14)^21^, human colon adenocarcinoma cell line (LoVo)^22,23^. Therefore we further measured the cytotoxicity effect of the enzymatic oxidation products of spermine, H_2_O_2_ and acrolein on the tested cell lines. Both of the two metabolites exhibited a dose-dependent toxicity effect on all cell lines, and the toxicity effect on drug-resistant cell lines was much stronger than that on wild cell lines (Fig. 1E,F). These results suggest that the cytotoxicity activity of spermine and spermidine may result from the enzymatic oxidation products of amine oxidases in FBS.

Polyamine concentrations are high in growing tissues, for example, in tumors^34^. If polyamines accumulate excessively within cells or in the extracellular environment, they can be oxidatively deaminated by amine oxidases to generate toxic products, leading to cell death, necrosis or apoptosis^31,34^. In current case, cells grew in normal culture medium containing adscititious polyamines; no additional bovine serum amine-oxidase was added. If the stronger cytotoxicity of spermine and spermidine to drug-resistant cells resulted from the toxic oxidative products, the toxic products may be generated by high amount of amine oxidases in drug-resistant cells or by the amine oxidases in FBS. Spermine oxidase (SMOX) is a key enzyme in the process of spermine metabolism in cells, catalyzing spermine into spermidine. Thus, we firstly measured the amount of SMOX in different cells using SMOX ELISA kit. However, there is no significant difference in the expression of SMOX between sensitive and drug-resistant cancer cells (Fig. S2), which suggests that SMOX may be irrelevant to the stronger cytotoxicity of spermine and spermidine to drug-resistant cells.

### The generation of H_2_O_2_ in cell culture media containing different serum supplements

**Figure 3:**
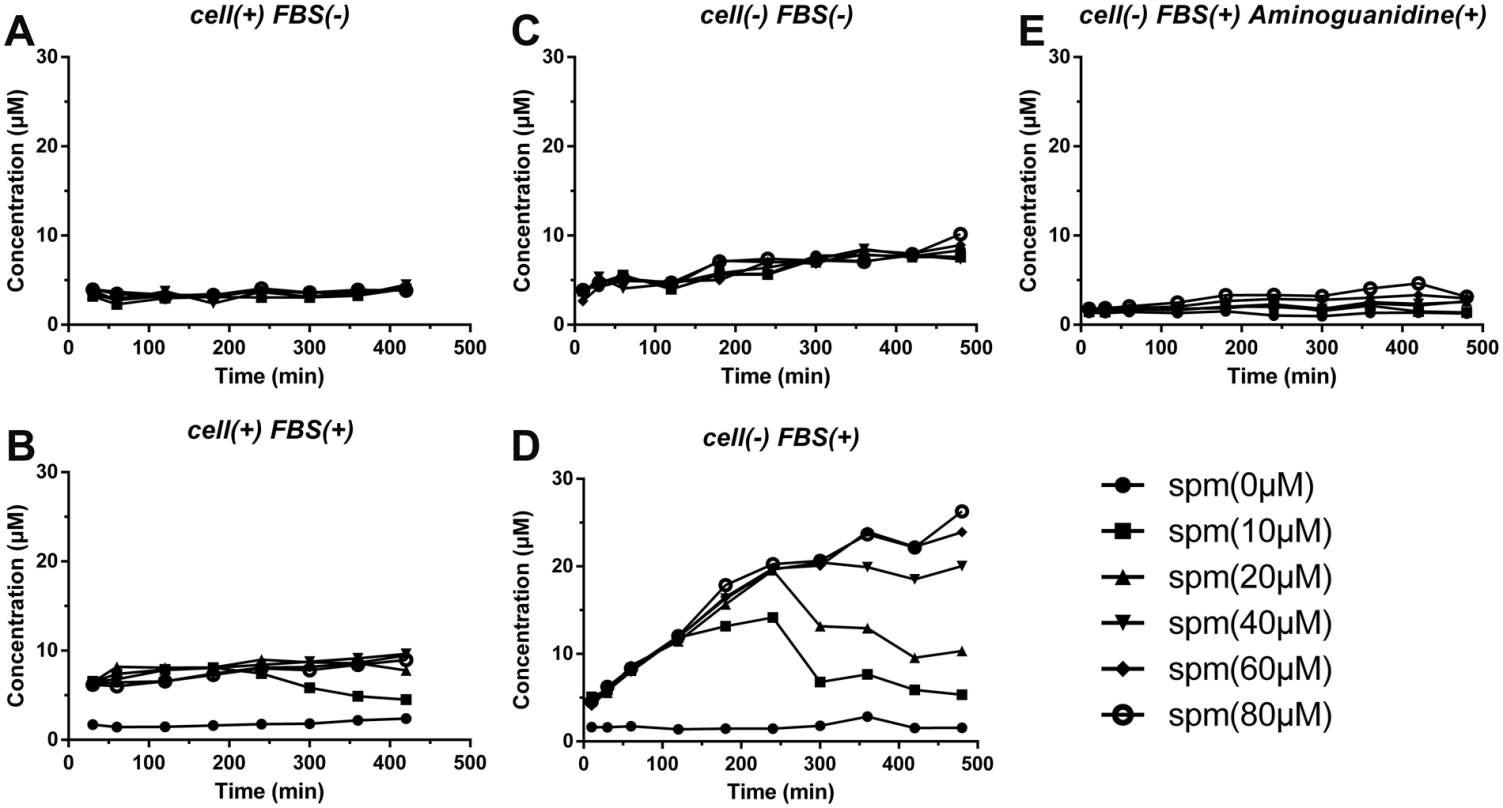
The generation of H_2_O_2_ in culture media after adding different concentrations of spermine (spm). The culture media containing (**A**) without FBS and with A549T cells, (**B**) with 10% FBS and A549T cells, (**C**) without FBS and cells, (**D**) with 10% FBS and without cells, (**E**) with 10% FBS and Aminoguanidine.

In order to demonstrate that the oxidation products of spermine were generated by the amine oxidases in serum, one of the metabolites of polyamines, hydrogen peroxide, was measured. The experiments were performed by incubating different concentrations of spermine in cultured medium in the absence or presence of 10% FBS and/or cells, and then measuring the concentrations of H_2_O_2_ at different incubation time. As shown in Fig. 3A–D, whether or not cells existing, after adding different concentrations of spermine, almost no H_2_O_2_ was detected in culture media without FBS (Fig. 3A,C); whereas H_2_O_2_ was detected in the media containing FBS, and the concentration of H_2_O_2_ changed with the incubation time and the amount of added spermine (Fig. 3B,D). These results suggest that H_2_O_2_ was generated by the amine oxidases in FBS. In order to further confirm our conclusion, aminoguanidine^35,36^, an inhibitor of serum amine oxidases, was added to the culture media containing FBS. As shown in Fig. 3E, almost no H_2_O_2_ was detected no matter how much spermine added, which confirmed that spermine was oxidated by serum amine oxidases in FBS. In addition, in the presence of FBS and spermine, high concentration of spermine (≥40 μM) caused continuously accumulation of H_2_O_2_ in media as incubation time increased; while low concentration of spermine (10 μM) caused H_2_O_2_ first increase (before 120 min) and then decrease (Fig. 3D), which may due to the exhaustion of low concentration of spermine in ~120 min and the consumption of H_2_O_2_ through decomposing and reaction with certain components in media. The concentration of H_2_O_2_ in the media without cells was much higher than that in the media with cells existing, indicating that cells in the media could consume the H_2_O_2_ produced by oxidation of spermine (Fig. 3B,D).

**Figure 4:**
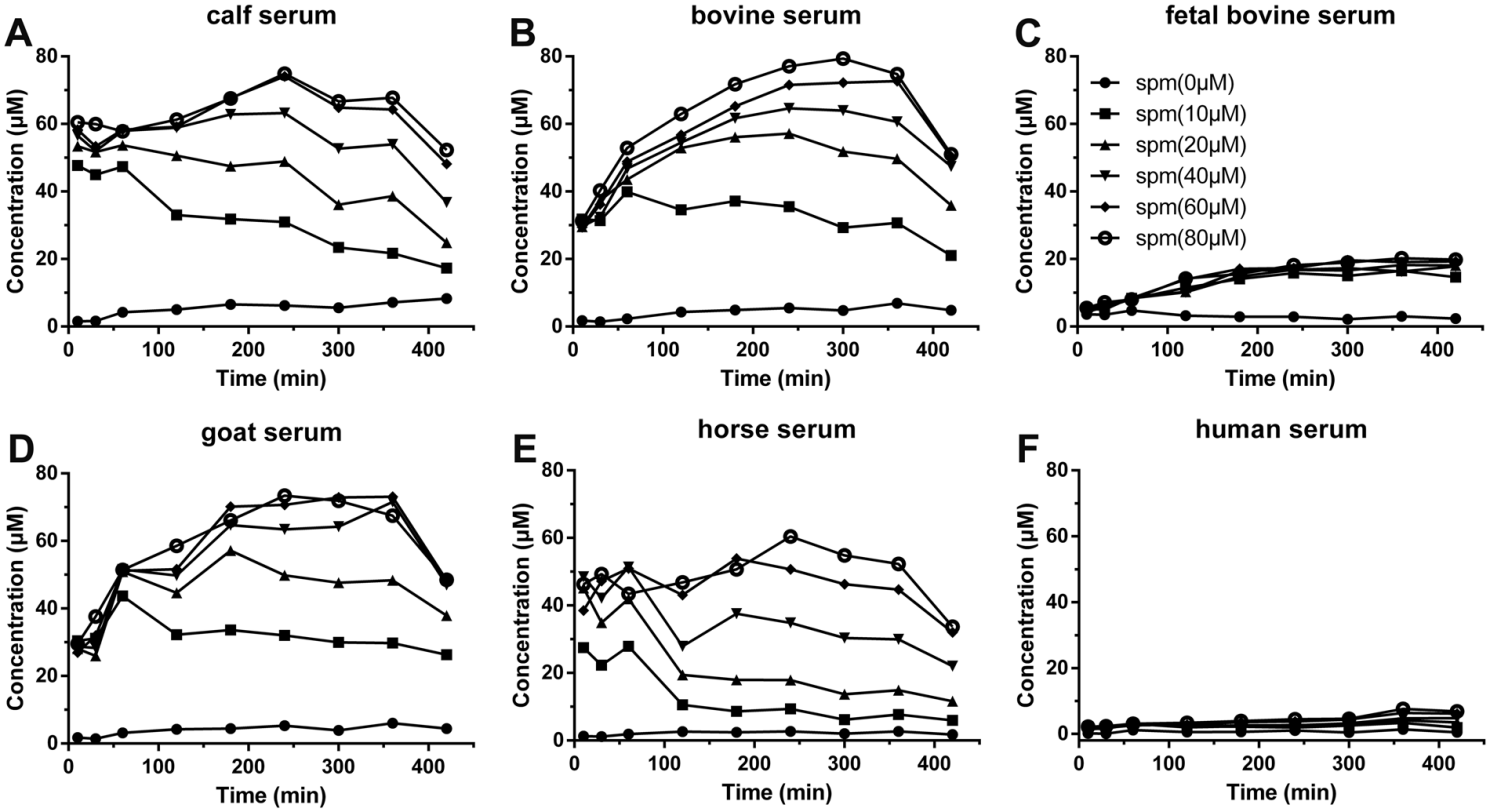
The generation of H_2_O_2_ in RPMI1640 medium with 10% different serums after adding different concentrations of spermine (spm).

To further investigate the oxidization ability of serums from different sources to polyamines, the generation of H_2_O_2_ in the media containing either human serum or other ruminant serums (including calf, bovine, goat and horse serums) was also tested. Interestingly, almost no H_2_O_2_ was detected in the media containing 10% or 50% human serum after adding spermine or spermidine (Figs 4, S3 and S4). While, after adding spermine, much higher concentrations of H_2_O_2_ were detected in the media containing 10% calf, bovine, goat and horse serum respectively than that in the media containing 10% FBS, and the same trend of first increase and then decrease was also observed. Similar results were also observed when adding spermidine into the media containing 10% of these serums (Fig. S3). Furthermore, higher concentration of serum was found to oxidize spermine with a higher speed. When adding spermine into the media containing 50% of these ruminant serums, higher concentrations of H_2_O_2_ were detected in the beginning and then decreased much faster than that in the media containing 10% serum (Fig. S4). These results suggest that a higher concentration of amine oxidases exists in calf, bovine, goat and horse serum than in FBS, and a very low amount of amine oxidases is present in human serum. Ruminant serum is the main source of serum supplement in cell culture because of the easy obtainment, the big difference in the components between ruminant and human serum may cause the misleading results in the studies of human cells.

In addition, the cytotoxicity assay of spermine and spermidine in the media containing 10% goat serum was performed. Both polyamines exhibited higher toxicity effects to all the tested cell lines than those measured in the media containing 10% FBS, which was consistent with the higher concentrations of H_2_O_2_ generated in the media containing 10% goat serum (Fig. S5). The drug-resistant cell lines (A549T and MCF-7R) showed much higher sensitivity to spermine and spermidine than their wild cell lines (A549 and MCF-7). These results further confirm that it is the oxidation products of polyamines caused their cytotoxicity to cultured cells.

### The mechanism investigation of the high sensitivity of drug-resistant cells to polyamines

**Figure 5:**
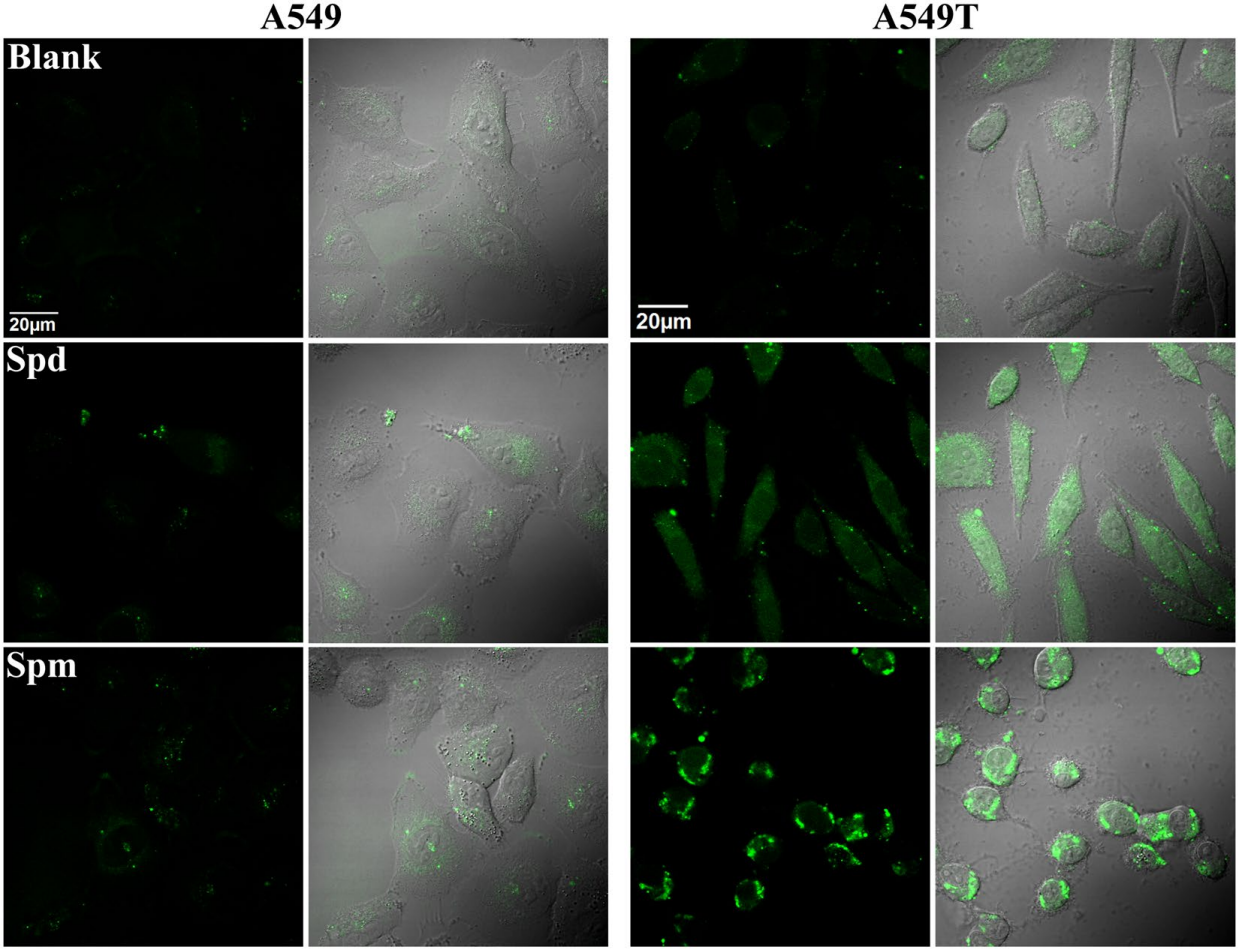
Confocal imaging of A549 and A549T cells stained with DCFH (10 μM) after treated with spermidine (Spd, 50 μM) and spermine (Spm, 50 μM) for 6 hours. Images were collected with excitation at 488 nm and emission at 500–550 nm, ×100 oil immersion lens.

In order to explain the higher sensitivity of drug-resistant cell lines to the cytotoxicity of polyamines than their wild cell lines, we tested the intracellular reactive oxygen species (ROS) by DCFHDA assay. The oxidization of DCFH (no fluorescence) by ROS produces DCF with high fluorescence in cells. As shown in Fig. 5, after treated with spermidine and spermine, the fluorescence intensity of A549T cells (drug-resistant cell type) was greatly increased, while the fluorescence increase in A549 cells was not significant; indicating that the concentration of ROS (mainly H_2_O_2_) in drug-resistant cells was much higher than that in the corresponding wild-type cells. This result suggests that the drug-resistant cells exhibit lower ability of scavenging ROS, which results in a much higher oxidative stress in cells.

**Figure 6:**
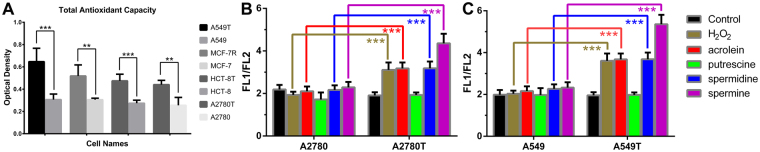
The total antioxidative capacity of different cells and the changes of mitochondrial membrane potential in cells treated with different compounds. Panel A is the total antioxidative capacity of eight cells was detected by ABTS^•+^ decolorization assay. Panel B and C are mitochondrial membrane potential changes in A2780 and A2780T, A549 and A549T cells respectively, were assessed using a mitochondrial membrane potential assay kit with JC-1, detected by Flow cytometry assay (FCM). The numbers of Optical Density are means ± SEM, n = 3–5 tests, **p < 0.01, ***p < 0.001, at least two independent experiments.

We further measured the total antioxidative capacity of the eight cell lines with a rapid ABTS^•+^ decolorization method. The antioxidants in cell contents could reduce the dark blue-green colored ABTS^•+^ radical to colorless ABTS form. As shown in Fig. 6A, the total antioxidative capacities of all the tested drug-resistant cell types were lower than their corresponding wild types, which resulted in the accumulation of H_2_O_2_ and the much higher oxidative stress in drug-resistant cells than the wild cell types. The lower antioxidative capacity of multidrug-resistant cancer cells has also been reported in a few papers^37^. This result can explain the selective cytotoxicity of spermine and spermidine to drug-resistant cells in media containing FBS.

Mitochondrial membrane potential (ΔΨ_m_) is considered as a potential target of ROS during oxidative stress-induced cell death^38^. The loss of ΔΨ_m_ is a hallmark of early cell apoptotic events. In order to understand of the different cytotoxicity of polyamines on drug-resistant cells and wild cells, we further compared the ΔΨ_m_ of two pairs of cells (A2780 and A2780T, A549 and A549T) after treated with spermine, spermidine, putrescine, H_2_O_2_ and acrolein. JC-1 dye exhibits potential-dependent accumulation in mitochondria, indicated by a fluorescence emission shift from green (FL1, ~529 nm, low ΔΨ_m_) to red (FL2, ~590 nm, high ΔΨ_m_) due to concentration-dependent formation of red fluorescent J-aggregates. It was used to measure the change of ΔΨ_m;_ the fluorescence ratio of FL1/FL2 can reflect the decrease in mitochondrial membrane potential. As shown in Fig. 6B,C, spermine, spermidine, H_2_O_2_ and acrolein but not putrescine significantly increased the FL1/FL2 of drug-resistant cell lines (A2780T and A549T), but did not change the FL1/FL2 of wild cell lines (A2780 and A549), indicating the loss of ΔΨ_m_ in drug-resistant cell lines was induced by spermine, spermidine, H_2_O_2_ and acrolein. Among these molecules, spermine caused a much higher increase of FL1/FL2. All these results are well consistent with the results of cytotoxicity assay, and further confirm that the oxidation products of spermine and spermidine caused high oxidative stresses in drug-resistant cells, resulting in the loss of ΔΨ_m_ and subsequent cell death.

In summary, we have tested the cytotoxicity of polyamines, including putrescine, spermidine, spermine and TETA. Spermidine and spermine showed high toxicity effect to drug-resistant cancer cell lines (A549T, MCF-7R, HCT-8T and A2780T) and low toxicity effect to the wild cell lines (A549, MCF-7, HCT-8 and A2780) (Fig. 1). Spermine was further demonstrated to significantly induce cell apoptosis and death in media containing FBS but not in FBS-free media, and cause S phase arrest of A549T cells (Fig. 2). Since the purified bovine serum amine oxidase was reported to oxide spermine causing the cytotoxicity to some cell lines^20,39^, the selective cytotoxicity of spermidine and spermine may be related to amine oxidases existed in FBS. Therefore, we tested the cytotoxicity of the enzymatic oxidation products of spermine, H_2_O_2_ and acrolein. H_2_O_2_ and acrolein showed similar toxicity effect on the tested drug-resistant cells but lower toxicity effect on wild cells. In order to demonstrate that spermidine and spermine were oxidated by amine oxidases in serum, we measured the generation of H_2_O_2_ after adding spermidine or spermine in cell culture media containing serum from different sources. After adding spermine or spermidine, a large amount of H_2_O_2_ was generated in cell culture media containing calf, bovine, goat or horse serums, a relatively low amount of H_2_O_2_ was generated in media containing FBS, and almost no H_2_O_2_ was detected in media containing human serum or in media without containing serum. The cytotoxicity of spermidine or spermine in media containing goat serum was stronger than that containing FBS. These results strongly confirmed that the cytotoxicity of polyamine resulted from the toxicity of oxidation products of polyamine generated by amine oxidases in ruminant serum supplements. There are a variety of oxidases that can degrade polyamines, such as diamine oxidase, spermine oxidase, acetylpolyamine oxidase, serum amine oxidase and others, unfortunately, the nomenclature of these enzymes is confusing^16^. Based on the substrate specificity to spermidine and spermine, in this case, the serum amine oxidase may play a vital role in degradation of polyamines. The higher sensitivity of the drug-resistant cancer cells to the cytotoxicity of spermine and spermidine was demonstrated to be the lower antioxidative capacity of drug-resistant cells than wild cells, which caused higher oxidative stresses and resulted in the loss of ΔΨ_m_ and subsequent cell death. Our results suggested that the enzymes in the serum supplement may cause unknown side effects on tested drugs. Therefore before drawing a conclusion of the drug effect on cells, the side effect from the serum supplement should be excluded firstly.

## Materials and Methods

### Chemicals

If not stated otherwise, chemicals were purchased from Sigma Chemical Co. (St. Louis, Mo.). Spermine oxidase Enzyme-Linked Immuno-Sorbent Assay (ELISA) kit, Jc-1, and Ampliflu^TM^ Red (AR) were purchased from Beyotime Biotechnology (Shanghai, China). Cell Counting Kit-8 (CCK-8), cell apoptosis kit and cell cycle kit were purchased from Dojindo chemical technology (Japan). Human, calf, bovine, goat and horse serum were purchased from Kete Biological Technology Co. Ltd (Jiangsu, China).

### Cell lines and cell culture

All cell lines used were of human origin. A549 (non-small cell lung cancer) and MCF-7 (breast cancer) cell lines were purchased from Cell Resource Center of Shanghai Institute for Biological Sciences (Chinese Academy of Sciences, Shanghai, China). A549T (Taxol-resistant A549 subline) and MCF-7R (Adriamycin-resistant MCF-7 subline) cell lines were from Shanghai Aiyan Biological Technology Co. Ltd (Shanghai, China). HCT-8 (colon carcinoma), A2780 (ovarian cancer), HCT-8T (Taxol-resistant HCT-8 subline) and A2780T (Taxol-resistant A2780 subline) were from Nanjing KeyGEN BioTECH Co. Ltd (Nanjing, China). PC-3 (prostate cancer) was from Typical Culture Preservation Commission Cell Bank, Chinese Academy of Sciences (Shanghai, China).

All cell lines were routinely cultured at 37 °C in humidified atmosphere with 5% CO_2_. Unless stated otherwise, the growth medium used for A549, A549T, MCF-7R, HCT-8, HCT-8T, PC-3 and A2780T cells was RPMI-1640 (Corning) containing 10% fetal bovine serum (FBS, Gibco) and 1% penicillin/streptomycin (Corning). MCF-7 and A2780 cells were propagated in Dulbecco’s Modified Eagle Medium (DMEM, Corning) with 10% fetal bovine serum (FBS, Gibco) and 1% penicillin/streptomycin (Corning). For comparison, some experiments replaced FBS with other serums (calf serum, bovine serum, goat serum, horse serum or human serum) and the same concentration.

### Cell proliferation assay

Cells were seeded in 96-well plates (Corning) at different densities according to their intrinsic growth rate. A549 and A2780 were seeded at a density of 1 × 10^5^/mL, MCF-7R, HCT-8, HCT-8T and A2780T were seeded at a density of 8 × 10^4^/mL, A549T and MCF-7 were seeded at a density of 5 × 10^4^/mL; and then all cells in the plates were incubated in a humidified atmosphere of 5% CO_2_ at 37 °C. After 24 h, spermine, spermidine, putrescine, triethylenetetramine, hydrogen peroxide and acrolein were added into each well at different concentration, respectively. The cells were further incubated for another 48 h without changing medium. Cell viability was measured by Cell Counting Kit-8 (CCK-8, Dojindo, Japan) according to the standard protocol outlined by the manufacturer. To ensure the results’ reliability, each concentration had two repeated wells and each experiment independently performed at least three times, and results are expressed as the mean of three experiments.

### Cell apoptosis analysis

A549T cells were seeded in a 6-well plate at a density of 1 × 10^5^/mL, after pre-incubation for 24 h, cells were added with spermine (0, 10, 20, 30, 50 and 80 μM) and incubated for 6 h in RPMI1640 with FBS or without FBS. Then cells collected and labeled according to the standard protocol of Annexin V-FITC Apoptosis Detection Kit (Beyotime Institute of Biotechnology) and detected by flow cytometer with FL1 and FL2 channels.

### Cell cycle measurements

A549T cells were seeded at a density of 1 × 10^5^/mL in a 6-well plate, after cultured for 24 h, spermine (0, 30 and 60 μM) were added into each well respectively and continuously incubated for 4 h. Then cell samples were prepared according to the protocol of Cell Cycle and Apoptosis Analysis Kit (Beyotime Institute of Biotechnology). Briefly, cells detached by 0.25% (w/v) trypsin-0.53 mM EDTA solution and fixed by 500 μL of 78% ethanol and stored at −20 °C over 24 h. The cells were added by 400 μL of PI and incubated at 37 °C for 30 min in the dark. The DNA content of cells was acquired by a FACScalibur flow cytometer (Becton Dickinson, USA) with FL2 channel. The data were analyzed by FlowJo software (Treestar, San Caros, USA).

### Detection of H_2_O_2_

In this assay, HRP catalyzed the decomposition of H_2_O_2_ causing the oxidation of Ampliflu^TM^ Red (AR) and resulting in the formation of resorufin, a highly fluorescent product. Under conditions where the molar ratio of AR to H_2_O_2_ was greater than five, resorufin was generated in a 1:1 stoichiometry with H_2_O_2._ To prepare the calibration curve, H_2_O_2_ was diluted to 0.391, 0.781, 1.563, 3.125, 6.25, 12.5, 25, and 50 μM with phosphate buffer saline (PBS), then incubated with a reaction mixture containing 10 μM AR and 0.1 unit/mL HPR for 20 min at 37 °C in a 96-well plate. Then the plate was read with a fluorescence microplate reader with excitation of 560 nm and emission at 595 nm. For samples, firstly, different concentrations of spermine or spermidine (0, 10, 20, 40, 60 and 80 μM) were incubated with different serums (calf serum, bovine serum, fetal bovine serum, goat serum, horse serum and human serum) at 37 °C; and the concentration of serums was 50% or 10%. 200 μL of samples were took out at different time (10, 30, 60, 120, 180, 240, 300, 360, 420 and 480 min), and incubated with 10 μM AR and 0.1 unit/mL HPR for 20 min at 37 °C in a 96-well plate, and the fluorescence intensity was measured with a microplate reader (Spectra Max M5, Molecular Device Co., USA).

### Reactive oxygen species detection in cells

ROS was measured by a reactive oxygen assay kit using 2′,7′-dichlorodihydrofluorescein diacetate (DCFH-DA) as the fluorescence probe. For ROS imaging in cells, cells (A549 and A549T) were seeded at desired concentration in covered glass-bottomed confocal dishes (NEST Biotechnology Co. Ltd.), after cultured for at least 24 h, cells were washed once and incubated with spermidine (Spd, 50 μM) and spermine (Spm, 50 μM) in RPMI1640 containing 10%FBS for 6 hours, then cells were washed and incubated with DCFH-DA (10 μM) in RPMI1640 containing 10%FBS for 30 min. After washing with PBS, confocal imaging was performed on an OLYMPUS FV1000-IX81 confocal microscope (Olympus Corporation, Japan). Confocal images were processed by Olympus FV10-ASW 4.2 viewer software.

### Total antioxidative capacity assay with ABTS^•+^

2,2′-azino-bis (3-ethylbenzothiazoline-6-sulfonic acid) diammonium salt radical cation (ABTS^•+^) is a stable free radical frequently used for estimating the total antioxidative capacity (TAC) of natural products^40^. The antioxidative capacity of cell contents was measured by ABTS^•+^ decolorization assay^41^. Antioxidants in the sample reduced dark blue-green colored ABTS^•+^ radical to colorless ABTS form. The absorbance change at 414 nm was related to the total antioxidant level of the sample.

1 × 10^6^ cells was ultrasonically broken in 200 μL of cold Phosphate Buffer Solution (PBS) to release antioxidants, and centrifuged at 12000 g at 4 °C for 5 mins. 10 μL of the supernatant, and 200 μL of ABTS working solution (ABTS (10 mM) 2 μL, H_2_O_2_ (10 mM) 2 μL, and 196 μL PBS) were mixed in each well of 96-well plates and incubated for 10 mins at room temperature. 10 μL of ultrapure water was used as blank control. The absorbance at 414 nm was measured by a microplate reader (Spectra Max M5, Molecular Device Co.). The absorbance in each well reflected the total antioxidative capacity of each cell sample.

### Measurement of the change of mitochondrial membrane potential

2 × 10^5^ cells (A2780, A2780T, A549 and A549T cells) were seeded in 6-well plate, respectively. After 24 hours, the medium in each well was changed to fresh medium with 50 μM hydrogen peroxide, 70 μM acrolein, 50 μM putrescine, 50 μM spermidine or 50 μM spermine. After cultured for another 8 h, cells were harvested, stained with JC-1 probe for 20 mins at 37 °C, then washed twice with PBS, and measured by Flow cytometry (FCM).

## Electronic supplementary material

Supplementary Information
