# Supplementary material for: Oxidative degradation of polyamines by serum supplement causes cytotoxicity on cultured cells
Source: Sci Rep. 2018 Jul 10;8:10384. doi: 10.1038/s41598-018-28648-8 (PMC6039494; doi:10.1038/s41598-018-28648-8)
Supplement: Supplementary file 1 — Supplementary Information [file 41598_2018_28648_MOESM1_ESM.pdf]

Supporting Information:

Oxidative degradation of polyamines by serum supplement causes cytotoxicity on cultured cells

Linlin Wang[1,2], Ying Liu[1,2], Cui Qi[3], Luyao Shen[1,2], Junyan Wang[1,2], Xiangjun Liu[1,2], Nan Zhang[1,2,]*, Tao Bing[1,2], Dihua Shangguan[1,2,]*

[1] Department Beijing National Laboratory for Molecular Sciences, Key Laboratory of Analytical Chemistry for Living Biosystems, CAS Research/Education Center for Excellence in Molecular Sciences, Institute of Chemistry, Chinese Academy of Sciences, Beijing, 100190, China.

[2] University of the Chinese Academy of Sciences, Beijing, 100049, China.

[3] CAS Center for Excellence in Nanoscience, National Center for Nanoscience and Technology, Beijing, 100190, China

* Correspondence author: Tel&Fax: 86-10-62528509, E-mail: hszhang@iccas.ac.cn, sgdh@iccas.ac.cn

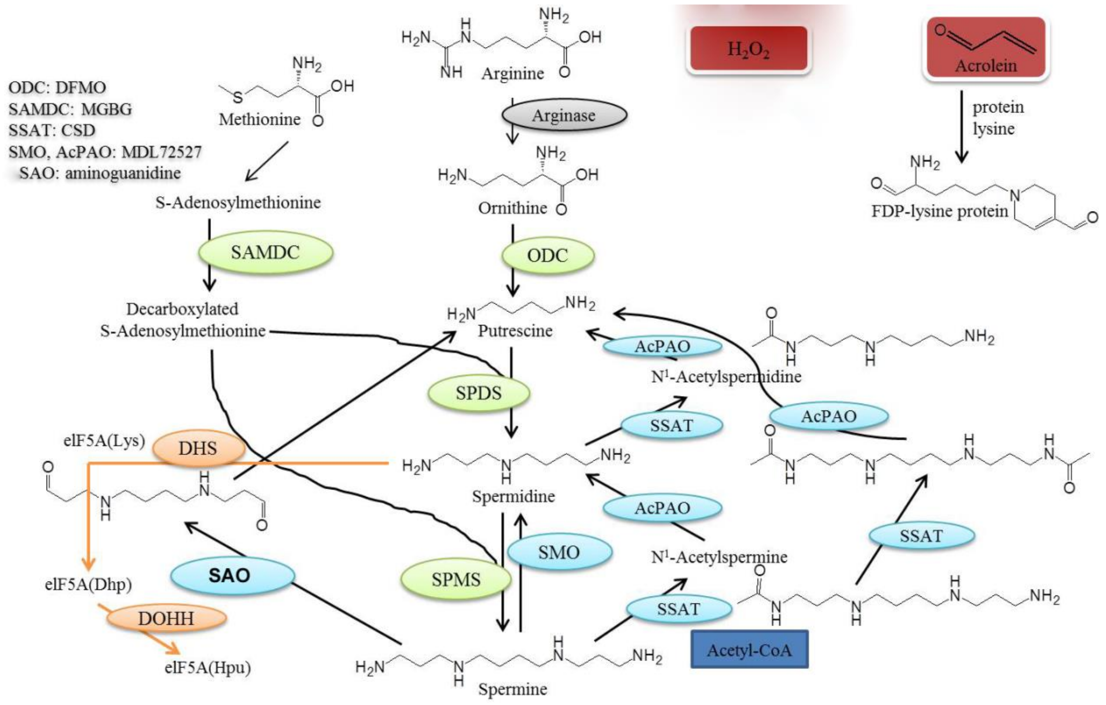

Figure S1. The scheme of spermine's bio-synthesis and metabolism.

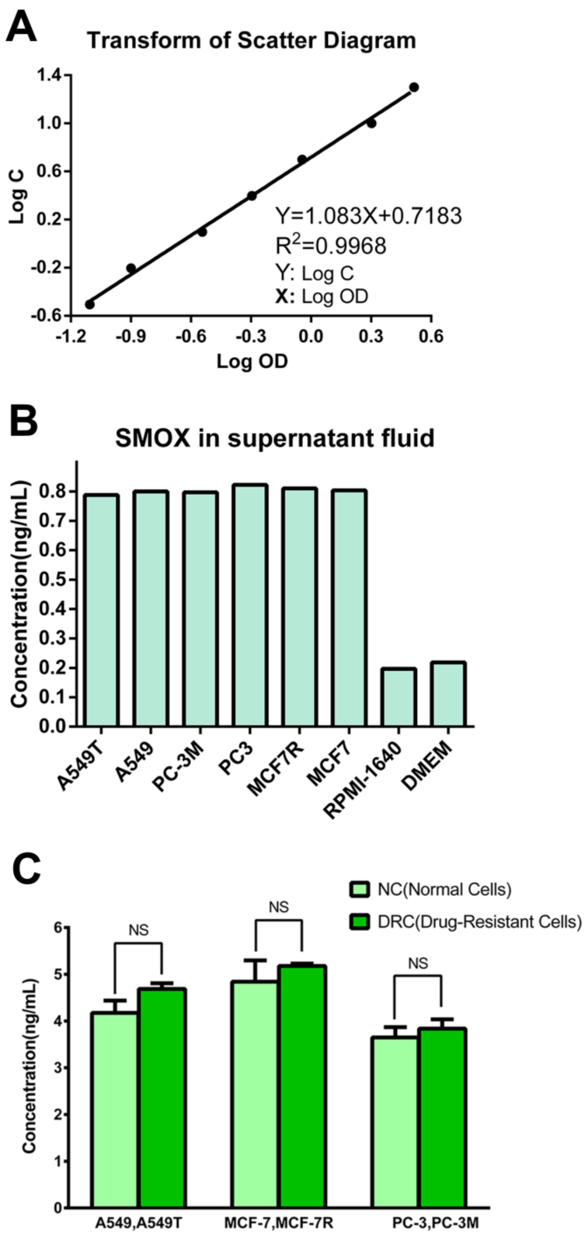

Figure S2. The content of SMOX in drug-resistant cells and sensitive cells. (A) the standard curve of SMOX; (B) the SMOX in supernatant fluid of different cells; (C) the SMOX in the extract of different cells.

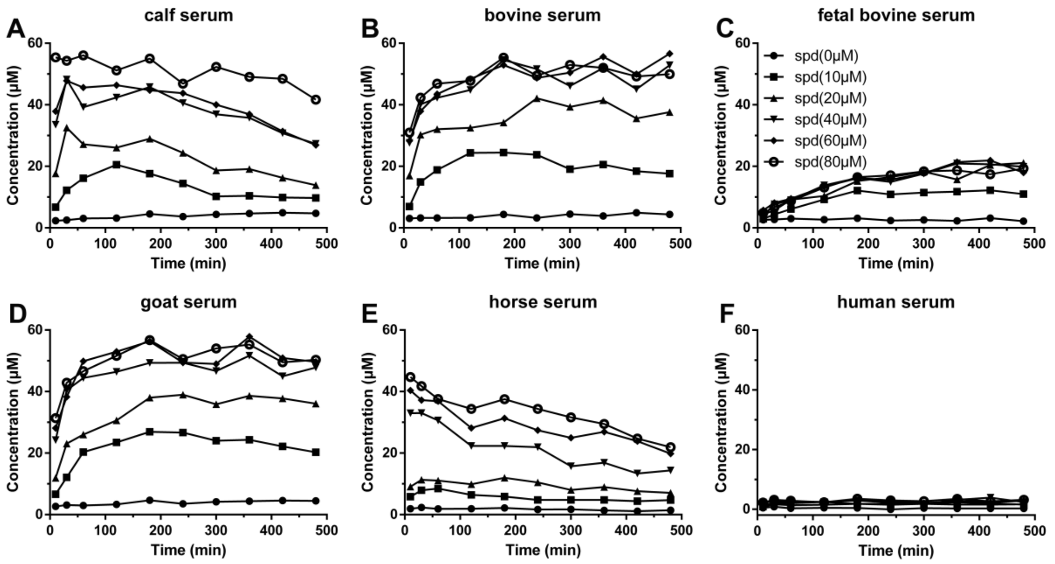

Figure S3. The generation of H_2O_2 in media containg 10% different serums after adding different concentrations of spermidine (spd).

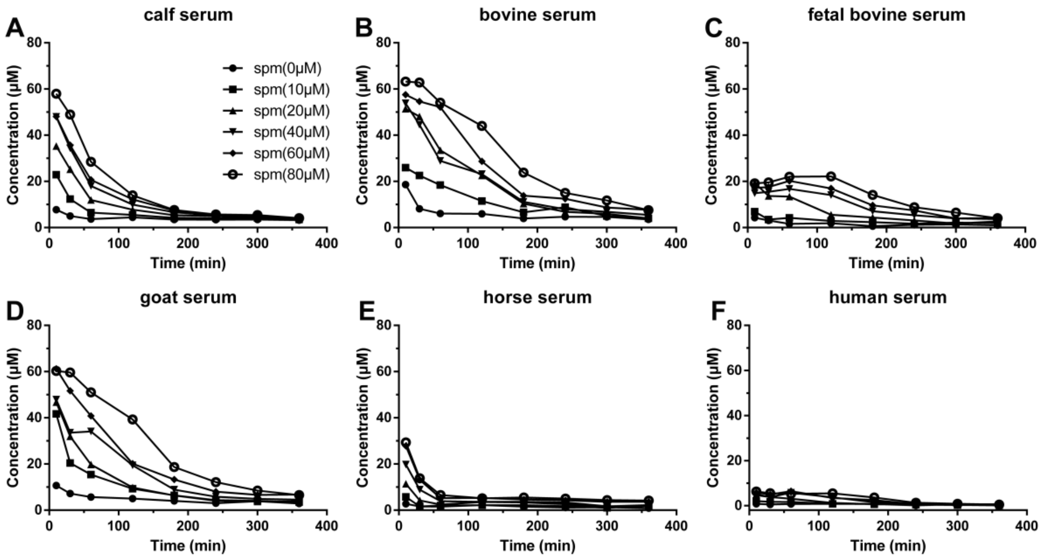

Figure S4. The generation of H_2O_2 in media containing 50% different serums after adding different concentrations of spermine (spm)

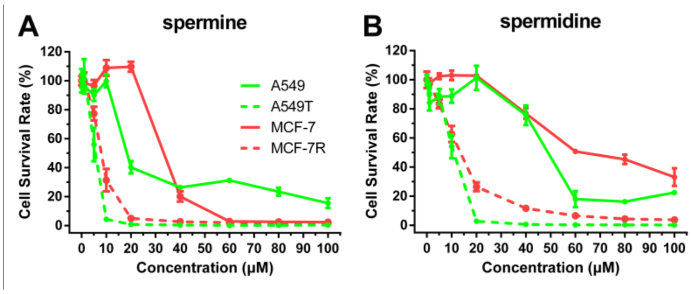

Figure S5. Dose-dependent cytotoxicity effects of spermine and spermidine in RPMI1640 containing 10% goat serum. A549, A549T, MCF-7 and MCF-7R cells were respectively incubated with different concentration of (A) spermine, (B) spermidine, at 37 ᵒC for 48h. Each point represents the mean value of the results of 2 to 4 wells of three or four experiments. The error bars indicate ±S. D. when not show, S. D. was smaller than the symbol.
